# Prognostic Factors for Mortality in Patients Aged 90 Years and Older with Proximal Femoral Fractures Undergoing Surgery: A Retrospective Study

**DOI:** 10.3390/jcm13247516

**Published:** 2024-12-10

**Authors:** Suguru Yokoo, Naofumi Shiota, Toru Sato, Sho Muguruma, Chuji Terada, Masanori Yorimitsu, Toshifumi Ozaki

**Affiliations:** 1 Department of Orthopaedic Surgery, National Hospital Organization Okayama Medical Center, Okayama 701-1192, Japan; naofumi@mx1.tiki.ne.jp (N.S.); sato.toru.ez@mail.hosp.go.jp (T.S.); s.muguo1@gmail.com (S.M.); 2 Department of Orthopaedic Surgery, Fukuyama City Hospital, Hiroshima 721-8511, Japan; ccterada@yahoo.co.jp; 3 Department of Musculoskeletal Traumatology, Faculty of Medicine, Dentistry, and Pharmaceutical Sciences, Okayama University, Okayama 700-8558, Japan; mayorimitsu@minos.ocn.ne.jp; 4 Department of Orthopaedic Surgery, Okayama University Graduate School of Medicine, Dentistry, and Pharmaceutical Sciences, Okayama 700-8558, Japan; tozaki@md.okayama-u.ac.jp

**Keywords:** body mass index, cognitive impairment, geriatric nutritional risk index, prognostic factor, proximal femoral fracture

## Abstract

**Background/Objectives:** Proximal femoral fractures (PFFs) among individuals aged ≥90 years are becoming more common with an aging population and are associated with high morbidity and mortality. This study analyzed the prognostic factors influencing survival in nonagenarian patients undergoing surgery for PFFs. **Methods:** We enrolled 285 patients who underwent surgery between 2016 and 2022. Patients were classified into two groups: those with postoperative survival >1 year (L) and those with postoperative survival ≤1 year (D). Factors assessed included age, sex, body mass index (BMI), cognitive impairment, fracture type, surgical timing, length of hospital stay, implant type, preoperative hemoglobin/albumin/white blood cell levels, and Geriatric Nutritional Risk Index (GNRI). **Results:** The mean age at surgery was 93.2 ± 2.8 years (mean follow-up = 18.9 months). The 12-month mortality rate was 28.8%. Intertrochanteric fractures were observed in 136/47 patients, and femoral neck fractures were observed in 67/35 patients in the L/D group, respectively (*p* = 0.13). Days from admission to surgery were not significantly associated with mortality (*p* = 0.56). The mean hospital stay was 17/22 days in the L/D group, respectively. Univariate analysis identified age, BMI, cognitive impairment, albumin level, and GNRI as statistically significant predictors. Multivariate analysis revealed length of hospital stay (odds ratio [OR] = 1.048 [95% confidence interval (CI): 1.019–1.078]; *p* = 0.001), cognitive impairment (OR = 3.082 [95% CI: 1.367–6.945]; *p* = 0.007), and GNRI (OR = 0.929 [95% CI: 0.901–0.958]; *p* < 0.001) as independent predictors of mortality. **Conclusions:** This study identified cognitive impairment, a low GNRI, and prolonged hospital stay as independent prognostic factors for 1-year mortality in nonagenarian patients with PFFs. These findings highlight the importance of addressing malnutrition and cognitive decline through tailored interventions, alongside optimizing surgical timing and hospital care. A multidisciplinary approach remains essential for improving survival outcomes in this vulnerable population.

## 1. Introduction

Proximal femoral fractures (PFFs) are prevalent among older adults and pose significant clinical challenges. With an aging society, the occurrence of PFFs among nonagenarians has become increasingly common. Japan is among the most rapidly aging countries globally, with >10% of its population aged ≥80 years [1,2]. As populations age, the incidence of PFFs is expected to increase [3,4]. Consequently, research on the prevention, treatment, and prognosis of PFFs is becoming increasingly important [5,6].

A previous study [7] found that individuals aged ≥85 years have a 15–18-fold higher risk of hip fracture than do those aged 50–54 years. Another study [8] reported that the incidence of PFFs among individuals aged ≥90 years is eight times higher than that among those in their seventies. Previous studies [9,10] have shown that PFFs are associated with high mortality rates and significant morbidity.

The risk of PFFs increases with age, primarily due to osteoporosis, making these fractures a significant cause of morbidity and mortality in advanced age [11]. Hip fractures profoundly impact the function and quality of life of patients aged ≥90 years. While surgical intervention and early rehabilitation remain the standard treatment for PFFs, limited data exist on surgical outcomes in nonagenarians, leaving many aspects of their prognosis unclear [12].

Multidisciplinary approaches play a crucial role in optimizing outcomes in older patients with hip fractures. Integrated orthogeriatric treatment models—involving surgeons, geriatricians, physiotherapists, and nutritionists—have been shown to reduce complications and improve mortality rates in older populations [13,14]. Given the unique physiological and psychological challenges faced by nonagenarians, including higher rates of comorbidities and complications, a collaborative approach tailored to their needs is essential for improving survival and recovery [13].

Mortality after hip fracture surgery is influenced by multiple factors, including age, time to surgery, sex, a high American Society of Anesthesiologists (ASA) score, cognitive impairment, and malnutrition [11,12,15]. Various scoring systems have been developed to predict postoperative outcomes in older patients with hip fractures. Among these, the Nottingham Hip Fracture Score is widely used to estimate the risk of mortality, particularly within the first 30 days after surgery. This score incorporates variables such as age, sex, preinjury mobility, and comorbidities to provide a comprehensive assessment of patient risk [16].

Nutritional status, as assessed using tools such as the Geriatric Nutritional Risk Index (GNRI) and cognitive impairment are particularly critical determinants of postoperative outcomes in older populations. The GNRI provides a comprehensive evaluation of nutritional status by incorporating both serum albumin levels and body mass index (BMI), which are independently associated with mortality in older adults with hip fractures. Recent studies, including a meta-analysis, have validated the predictive value of the GNRI for postoperative complications, highlighting its potential as a reliable prognostic tool [17]. Similarly, cognitive impairment is a well-recognized risk factor for poor surgical outcomes, including increased mortality and reduced functional recovery. A recent systematic review has revealed that dementia significantly contributes to early mortality after hip fracture surgery, although it primarily focused on younger older adult populations overall [15]. While prior studies have examined hip fracture outcomes in nonagenarians, many lacked using standardized cognitive scoring methods, which may underestimate the true impact of cognitive impairment [13]. The use of validated tools, such as the Abbreviated Mental Test Score (AMTS), provides a more objective and reliable assessment of cognitive status, enabling a clearer understanding of its influence on mortality.

Most existing studies on hip fractures in nonagenarians have focused on short-term outcomes, such as 30- or 90-day mortality, with limited attention to longer-term survival rates [18,19,20]. For example, while several studies have highlighted the immediate risks associated with surgery, few have systematically evaluated 1-year survival rates or the factors influencing them. Furthermore, while 1-year mortality data are relatively abundant for younger older populations, such as those in their seventies or eighties, these data are scarce for nonagenarians. Given the unique vulnerabilities of this age group, understanding 1-year outcomes is critical for guiding treatment strategies and improving prognostic predictions. We conducted this study to fill this gap by analyzing the 1-year survival outcomes and their associated prognostic factors in nonagenarian patients with PFFs.

Despite the identification of these risk factors, a consensus on the most predictive factors of mortality in patients aged ≥90 years with PFFs remains elusive. In this study, we aimed to analyze the surgical outcomes for PFFs in patients aged ≥90 years and to identify factors associated with their survival.

## 2. Materials and Methods

### 2.1. Ethics

The study was conducted in accordance with the Declaration of Helsinki and approved by the Institutional Review Board of the National Hospital Organization Okayama Medical Center (approval number: 2022-154; approval date: 20 December 2022). Informed consent was obtained from all subjects involved in the study.

### 2.2. Study Design and Participants

A total of 305 consecutive patients aged ≥90 years who underwent surgery for PFFs at our hospital between January 2016 and December 2022 were retrospectively reviewed. After excluding 20 patients because of insufficient follow-up, 285 patients were included in the study. Patients with postoperative survival >1 year were categorized as the L group, while those with postoperative survival ≤1 year were categorized as the D group. The exclusion criteria included fractures related to bone tumors and patients who had undergone osteosynthesis revision.

### 2.3. Operative Procedure

All patients underwent surgery under spinal or general anesthesia. Intertrochanteric fractures were treated using a Dynamic Hip System (DePuySynthes, Warsaw, IN, USA) or an intertrochanteric nail. The intertrochanteric nails used were the TFN-ADVANCED™ Proximal Femoral Nailing System (DePuySynthes) or the Unicorn nail (HOYA Technosurgical, Tokyo, Japan). The choice of implant was based on patient factors such as bone quality, fracture stability, and overall health status. Non-displaced femoral neck fractures were treated with cannulated cancellous hip screws, while displaced femoral neck fractures were managed with hemiarthroplasty. All patients were allowed full weight-bearing ambulation postoperatively.

### 2.4. Follow-Up

Hospital transfer was typically scheduled for approximately 14 days postoperatively, depending on patient progress. After transfer, patients were monitored at the outpatient clinic at 12 weeks, 6 months, 12 months, and annually thereafter—or until death. Survival status was ascertained through hospital follow-up visits or by contacting patients and their families. Mortality at 6 months, 12 months, and at the end of the study period was documented. Patients who survived were followed up for ≥12 months, while those who passed away were followed up until the date of death, which marked the endpoint for mortality analysis. Thus, the follow-up duration ranged from the date of surgery to death or the conclusion of the study. The primary endpoint was either death or the conclusion of the study (December 2023).

### 2.5. Determination of Prognostic Risk Factors

Factors evaluated included age; sex; BMI; cognitive impairment; fracture type; days from injury to surgery; days from admission to surgery; length of hospital stay; duration of surgery; levels of hemoglobin, albumin, and white blood cells; and Geriatric Nutritional Risk Index (GNRI) on admission. The GNRI was calculated based on serum albumin levels and body weight relative to ideal body weight using the following equation:

GNRI = (1.489 × serum albumin (g/dL)) + (41.7 × actual body weight (kg)/ideal body weight (kg))(1)

where ideal body weight was defined as the weight corresponding to a BMI of 22 kg/m^2^. This index is commonly used to assess nutritional risk in older patients [21]. Data were collected by reviewing hospital records and interviewing patients and their relatives. Cognitive impairment was assessed using the AMTS, with a threshold of 7 points (with scores of ≤6 indicating impairment). Prefracture functional status was assessed and categorized into five groups based on the degree of mobility: patients who could walk independently without assistive devices (Free Walk), those who required a cane (Cane Gait), those using a walker (Walker), and those who were either wheelchair-bound (Wheelchair) or bedridden (Bedridden). For further analysis, these categories were consolidated into two groups: those who were able to walk (Free Walk, Cane Gait, or Walker) and those who were unable to walk (Wheelchair or Bedridden).

### 2.6. Statistical Analysis

Differences in patient characteristics were assessed using the chi-square test for categorical variables and the unpaired *t*-test for continuous variables. Post hoc power analysis was conducted following the univariate analysis to ensure that the study had sufficient statistical power to detect significant differences, with a threshold set to 0.80. Multivariate analysis was subsequently performed using logistic regression to identify independent factors associated with mortality. Receiver Operating Characteristic (ROC) curve analysis was employed to determine the optimal cut-off values for statistically significant variables in the univariate or multivariate analyses. The Youden index was calculated to identify cut-off values that maximized the balance between sensitivity and specificity, and the area under the curve (AUC) was used to assess predictive accuracy. Patient survival was analyzed using the Kaplan–Meier method, and between-group differences in survival were assessed using the log-rank test. Statistical significance was set at *p* < 0.05. All statistical analyses were conducted using GraphPad Prism (version 8.0; GraphPad Software, San Diego, CA, USA).

## 3. Results

**Figure 1:**
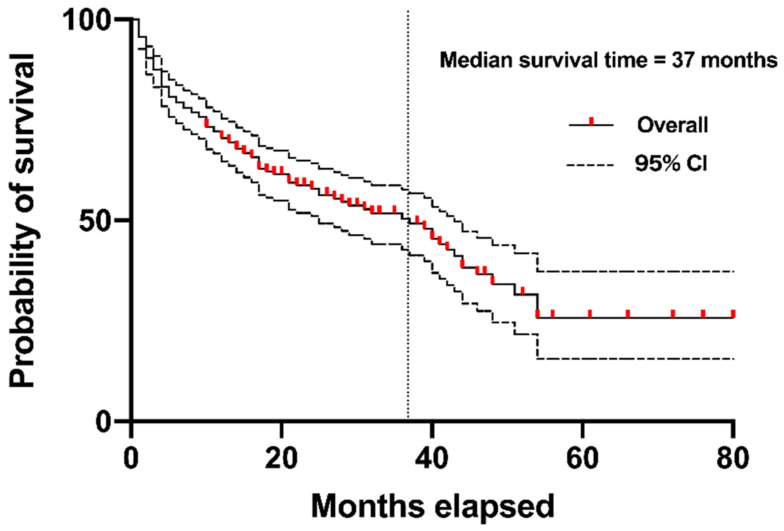
Kaplan–Meier curve for overall survival illustrating the probability of survival following surgical intervention for proximal femoral fractures. The solid line represents the Kaplan–Meier curve. The dashed lines represent the 95% confidence intervals. The median survival time was 37 months, with a 95% confidence interval ranging from 29.0 to 44.5 months. The mortality rates were 1.4% (*n* = 4), 18.9% (*n* = 54), and 28.8% (*n* = 82) at 1 month, 6 months, and 12 months after surgery, respectively. The curve represents long-term survival outcomes up to approximately 8 years.

The mean age at surgery was 93.2 ± 2.8 years. The mean follow-up period was 18.9 (range = 0–80) months (Table 1). The cohort comprised 43 males and 242 females. The numbers of deaths within 1 month, 6 months, and 12 months after surgery were 4 (1.4%), 54 (18.9%), and 82 (28.8%), respectively. According to the Kaplan–Meier method, the median survival time was 37 months (Figure 1). The survival probability declined steeply during the first year after surgery, with mortality rates of 1.4%, 18.9%, and 28.8% at 1 month, 6 months, and 12 months, respectively.

A total of 183 patients had intertrochanteric fractures; 102 had femoral neck fractures (Table 2). Among the patients with intertrochanteric fractures, 94 and 84 were treated with the Dynamic Hip System and intertrochanteric nails, respectively. Among the patients with femoral neck fractures, 28 were treated with cannulated cancellous hip screws, while 74 underwent hemiarthroplasty. The L group (patients who survived >12 months after surgery) included 136 and 67 patients with intertrochanteric and femoral neck fractures, respectively. The D group (patients who died within 12 months after surgery) included 47 and 35 patients with intertrochanteric and femoral neck fractures, respectively (*p* = 0.13).

The mean surgical duration was 82.3 ± 29.7 min for the L group and 80.2 ± 26.1 min for the D group (*p* = 0.77). The mean duration from injury to surgery was 3.0 ± 4.0 days; the mean duration from admission to surgery was 1.9 ± 2.6 days; and the mean length of hospital stay was 18.9 ± 9.3 days. The mean durations from injury to surgery were 3.1 ± 4.2 and 2.7 ± 3.5 days in the L and D groups, respectively (*p* = 0.50). The mean durations from admission to surgery were 2.0 ± 2.7 and 1.9 ± 2.4 days in the L and D groups, respectively (*p* = 0.56). The mean lengths of hospital stay were 17 ± 7.8 and 22 ± 11.7 days in the L and D groups, respectively (*p* = 0.003) (Table 2).

### 3.1. Univariate Analysis of Prognostic Factors

Other examined variables included age (mean = 92.8 years in the L group and 94.0 years in the D group; *p* = 0.015); sex (male/female = 26/177 in the L group and 17/65 in the D group; *p* = 0.10); BMI (mean = 20.2 kg/m^2^ in the L group and 18.7 kg/m^2^ in the D group; *p* < 0.0001); cognitive impairment (69.5% in the L group and 89.0% in the D group; *p* = 0.0004); prefracture functional status (ambulatory patients = 86.2% in the L group and 81.7% in the D group; *p* = 0.2261); hemoglobin level on admission (mean = 10.8 g/dL in the L group and 10.7 g/dL in the D group; *p* = 0.51); albumin level on admission (mean = 3.6 g/dL in the L group and 3.3 g/dL in the D group; *p* < 0.0001); white blood cell count (mean = 9.1 µL in the L group and 9.0 µL in the D group; *p* = 0.62); GNRI on admission (mean = 91.8 in the L group and 84.9 in the D group; *p* < 0.0001); and implant type (Dynamic Hip System/intertrochanteric nail/cannulated cancellous hip screws/hemiarthroplasty = 69/67/18/49 in the L group and 25/22/10/25 in the D group; *p* = 0.48) (Table 2). In the univariate analysis, age, BMI, length of hospital stay, cognitive impairment, albumin level on admission, and GNRI were found to be significantly associated with overall survival. Post hoc power analyses were conducted to confirm the adequacy of the sample size for these key variables. The power achieved for each variable was as follows: age (0.7661), BMI (0.9826), length of hospital stay (0.9156), cognitive impairment (0.9858), albumin level (0.982), and GNRI (0.9998). The results demonstrated sufficient statistical power for most variables, except for age, which had a slightly lower but still acceptable power.

### 3.2. Multivariate Analysis of Prognostic Factors

Multivariate analysis demonstrated that the length of hospital stay (odds ratio [OR] = 1.048 [95% confidence interval (CI): 1.019–1.078]; *p* = 0.001), cognitive impairment (OR = 3.082 [95% CI: 1.367–6.945]; *p* = 0.007), and GNRI (OR = 0.929 [95% CI: 0.901–0.958]; *p* < 0.001) were independently associated with mortality (Table 3).

Although the age, BMI, and albumin level variables were statistically significant in the univariate analysis, they were not retained in the final multivariate model following stepwise selection. This was attributed to their relatively small independent effects compared to other variables, such as the length of hospital stay, cognitive impairment, and GNRI, which were retained in the final multivariate model.

### 3.3. Kaplan–Meier Survival Analysis

**Figure 2:**
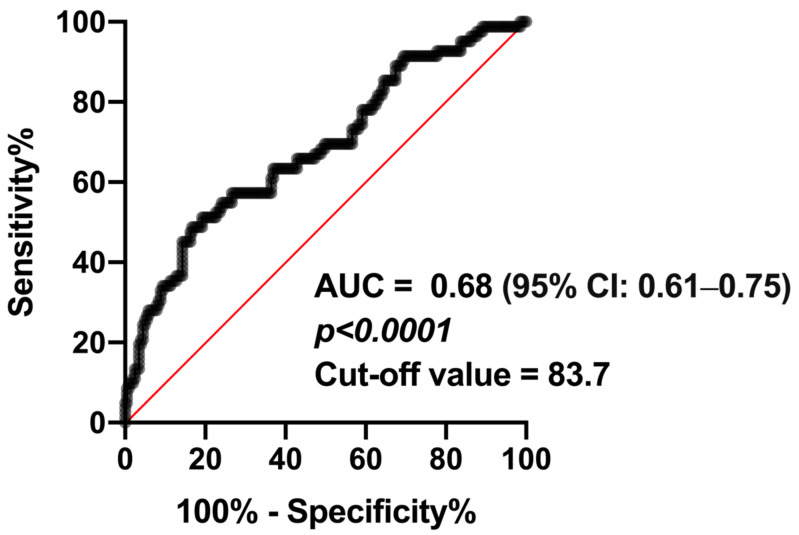
Receiver operating characteristic curve based on a sample size of 285 patients illustrating the diagnostic ability of the Geriatric Nutritional Risk Index in predicting survival outcomes. The area under the curve (AUC) was 0.68 (95% confidence interval [CI]: 0.61–0.75) (*p* < 0.0001). The Youden index cut-off value was 83.7.

Kaplan–Meier survival analysis was conducted to further explore the impact of the GNRI and cognitive impairment on patient survival. The GNRI cut-off value was determined using the ROC curve, which evaluates the diagnostic ability of the GNRI to predict survival outcomes. The area under the curve (AUC) was 0.68 (95% CI: 0.61–0.754, indicating moderate predictive accuracy. A Youden index cut-off value of 83.72 was established to optimally differentiate between higher- and lower-risk patients (Figure 2). This threshold reflects a balance between sensitivity and specificity, supporting its clinical utility in identifying patients at an elevated risk of poor outcomes.

**Figure 3:**
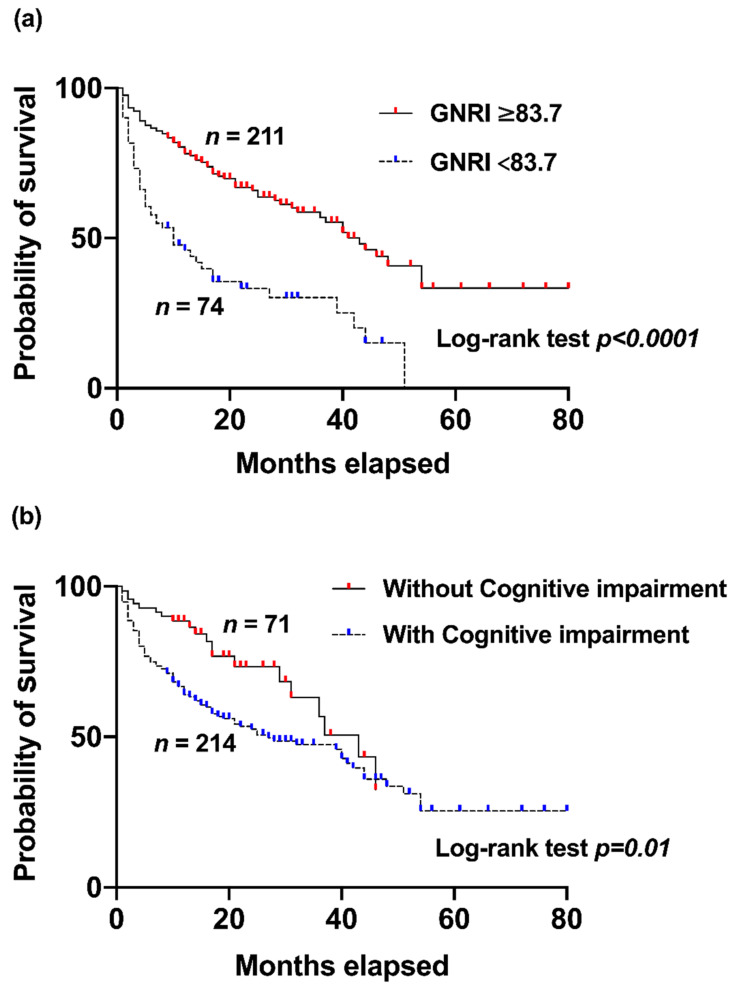
Kaplan–Meier survival curves for Geriatric Nutritional Risk Index (GNRI) and cognitive impairment: (**a**) Kaplan–Meier survival curves comparing patients with a higher (≥83.7) and lower (<83.72) GNRI. The survival probability is shown over the elapsed months, indicating that patients with a higher GNRI were found to have significantly better survival outcomes (*p* < 0.0001; log-rank test). (**b**) Kaplan–Meier survival curves comparing patients with and without cognitive impairment (defined as an Abbreviated Mental Test Score of 7). The analysis shows that patients without cognitive impairment were found to have significantly better survival outcomes compared to those with cognitive impairment (*p* = 0.01; log-rank test).

Additionally, patients were grouped based on cognitive impairment, which was defined by an AMTS score of 7. The Kaplan–Meier survival curves indicated that patients without cognitive impairment had a significantly higher survival probability than did those with cognitive impairment (*p* = 0.01; log-rank test) (Figure 3b).

## 4. Discussion

### 4.1. Key Prognostic Factors for Survival

This study identified several prognostic factors for survival in patients aged ≥90 years with PFFs, including BMI, cognitive impairment, albumin level, and GNRI. These findings highlight the importance of nutritional and cognitive assessments in this vulnerable population. The high post hoc power achieved for the key prognostic factors (age, BMI, cognitive impairment, albumin level, and GNRI) supports the reliability of the study’s findings. The powers for BMI, cognitive impairment, albumin level, and GNRI were all >0.98, indicating that the sample size was sufficient to detect significant differences in survival outcomes. Although the power for age was slightly lower at 0.7661, it was still within the acceptable limits for drawing conclusions.

### 4.2. Mortality and Hospital Stay

In this study, longer hospital stays, a lower BMI on admission, cognitive impairment, and lower albumin levels on admission were associated with higher mortality rates. Ko et al. [22] have reported that shorter hospital stays for older patients with hip fractures could be feasible without adversely affecting outcomes provided that tailored postoperative care has been implemented. Early surgery and rehabilitation are crucial for reducing mortality rates, whereas the length of hospital stay directly affects prognosis [9]. However, it is important to note that the length of hospital stay is influenced by institutional policies, social factors, and logistical constraints, including the availability of rehabilitation hospitals, which is particularly relevant in Japan where postoperative transfers are common. These factors may not directly reflect the patient’s clinical outcomes, highlighting the need to evaluate the length of hospital stay alongside other variables when assessing mortality risk.

### 4.3. Nutritional Status: GNRI, BMI, and the Obesity Paradox

Previous studies [10,15] have also demonstrated that both a low BMI and low serum albumin levels are independently associated with higher mortality rates in patients with PFFs. Given that the GNRI combines these two critical indicators of nutritional status, it provides a more comprehensive assessment of malnutrition and its impact on survival outcomes. However, to our knowledge, no previous studies have specifically examined the GNRI as a prognostic factor in nonagenarians with hip fractures. Our study is the first to demonstrate that a low GNRI is significantly associated with increased mortality in patients aged ≥90 years, highlighting the importance of addressing nutritional status in this highly vulnerable population. Our findings suggest that, similar to younger cohorts, malnutrition remains a critical determinant of survival, and interventions aimed at improving nutritional status could play a key role in improving outcomes for nonagenarians with hip fractures. Older patients with cognitive impairment have poorer outcomes after hip fracture surgery, including higher mortality rates and reduced functional recovery [23,24]. A multidisciplinary approach is essential for managing patients with dementia undergoing hip fracture surgery, focusing on tailored postoperative care to improve outcomes.

Hypoalbuminemia (low serum albumin levels) has been found to significantly increase postoperative complications, including infections, delayed wound healing, and prolonged hospital stays [25]. Preoperative nutritional status is crucial for determining patient outcomes, highlighting the importance of appropriate nutritional management and postoperative care strategies. Lower preoperative serum albumin levels are associated with higher postoperative mortality and morbidity rates [26,27]. Routine assessment of albumin levels as part of the preoperative evaluation could enhance long-term survival outcomes in this vulnerable patient population.

Previous studies have shown that a high BMI is associated with increased morbidity and mortality rates among older patients with PFFs. For instance, Soliman et al. [28] have highlighted higher rates of comorbidities and post-trauma complications in patients with obesity than in those with a normal weight. Similarly, another study [12] has emphasized the negative impact of a high BMI on patient outcomes owing to the increased risk of complications. However, the findings of the present study suggest that, in patients aged ≥90 years, a high BMI may be linked to better survival outcomes. This could be because of a better nutritional status and greater muscle mass, which are crucial for recovery in very old individuals. This “obesity paradox” indicates that a high BMI may provide a survival advantage in this age group by offering greater energy reserves during acute illness or injury. In contrast, a low BMI has been consistently associated with poorer surgical outcomes, including a higher postoperative mortality rate, delayed recovery, and an increased risk of complications. A low BMI often reflects malnutrition and sarcopenia, conditions that compromise the body’s ability to heal and recover after major trauma or surgery [29,30]. Malnutrition, in particular, has been linked to immune dysfunction, prolonged wound healing, and an increased susceptibility to infections, all of which can significantly impair recovery after hip fracture surgery. This highlights the importance of early nutritional assessment and intervention, especially in the oldest old population. Additionally, other studies [31,32] have indicated that a high BMI in later life may be associated with a lower risk of mortality from certain conditions, particularly cardiovascular disease. Individuals who entered adulthood with a normal BMI and gradually became overweight tended to live the longest [33]. These findings highlight the need for personalized medical approaches in treating very old patients with PFFs. While traditionally viewed as a negative factor, a high BMI might be beneficial for the oldest old, suggesting that clinical guidelines should be refined to account for these unique characteristics. Further research is needed to fully understand this association and its implications for patient care.

### 4.4. Surgical Timing and Patient-Specific Factors

Early surgery is expected to reduce mortality rates, enhance functional recovery, and decrease the risk of complications in patients with PFFs [34,35]. However, acute medical conditions, such as heart failure, pneumonia, severe coagulation disorders, and metabolic abnormalities, may contraindicate early surgery [12,36,37]. Stabilizing the overall condition of older patients with lower surgical tolerance before proceeding may be necessary. In the present study, the number of days from admission to surgery was not significantly associated with mortality (*p* = 0.56). This finding contrasts with those of previous studies suggesting that early surgery improves outcomes in patients with hip fractures [34,35]. One possible explanation for this discrepancy is that the relatively short time from admission to surgery (mean 2.0 days) in our cohort may have limited the ability to detect significant differences. Additionally, many patients experienced delays due to referral processes prior to hospital admission, which may have influenced the definition of “delayed surgery” compared to other studies. This highlights the importance of evaluating pre-hospital factors that could impact surgical timing and outcomes.

Furthermore, the lack of a significant association between implant type and survival outcomes aligns with the notion that patient-specific factors, such as nutritional status and comorbidities, play a more critical role in determining survival than does implant selection.

### 4.5. Mortality Trends and Prognostic Insights

While many reports have focused on the prognosis of patients with PFFs, few have specifically addressed patients aged >90 years. Previous studies [38,39,40,41] have indicated that the 1-year mortality rate for surgically treated patients aged >90 years ranges from 12.1% to 54.7%. In the present study, the 1-year mortality rate was 28.8%, which is consistent with these findings. Poor prognostic factors in the surgical management of PFFs in patients aged ≥90 years include a preoperative ASA score of 4, female sex, delayed surgery, and pre-existing heart disease and endocrinopathies. In the present study, no patients with an ASA score of 4 underwent surgery. Although no significant difference was found regarding sex, a trend toward higher mortality rates among males was observed. Generally, the mortality rate after PFFs is higher among men than women [42,43,44]. This may be partly explained by the generally shorter life expectancy among men [45].

Non-surgical management of PFFs in older individuals with frailty is associated with higher mortality rates [46]. Surgery for femoral neck fractures in patients aged >90 years significantly was found to improve survival rates compared to non-surgical treatment [47]. Timely surgery and proper preoperative management can significantly improve outcomes and reduce mortality in this population [11,48].

### 4.6. Study Strengths and Limitations

This study has several strengths. First, the treatment outcomes were derived from a single-center study, which ensured consistent treatment protocols, minimized potential biases often associated with multicenter studies, and allowed for homogeneity in surgical teams and postoperative care. This consistency has likely enhanced the internal validity of our findings. Second, this study included a large sample size of 285 patients aged ≥90 years with PFFs, which represent a patient demographic that is typically underrepresented in the existing literature. Lastly, the high follow-up rate of 93.4% (285/305 patients) minimizes the risk of follow-up bias and reinforces the robustness of the study’s findings.

The main limitations of this study include its retrospective design and potential bias in data collection. As a retrospective study, it is subject to inherent biases, such as selection bias and information bias, which may affect the accuracy of the results. The retrospective nature of this study also limits our ability to establish causal relationships between the identified prognostic factors and mortality outcomes. Although multivariate analysis was used to adjust for confounding factors, residual confounding cannot be completely ruled out. While the sample size is large for this specific age group, it remains limited compared to studies with a broader population, potentially affecting the generalizability of the results. In this study, diabetes and cognitive impairment were selected as the primary comorbidities for analysis, as these conditions have been identified in prior studies as key prognostic factors. However, other comorbidities, such as cardiovascular and renal diseases, were excluded owing to data limitations and the need to maintain statistical power. Similarly, ASA scores were not included in this study, as specific documentation regarding ASA classifications was unavailable retrospectively from the medical records. Retrospectively assigning ASA scores based on subjective judgments, particularly for borderline categories such as ASA 2–3, posed a substantial risk of bias. This limitation highlights the necessity for standardized and systematic recording of ASA scores in future research to ensure more rigorous and reliable prognostic evaluations. This limitation may restrict the generalizability of our findings. Future studies should include a broader range of comorbidities to provide a more comprehensive analysis. Variations in treatment protocols across different healthcare facilities could lead to inconsistencies in patient care and outcomes. Moreover, while the single-center design ensures consistency, it may limit the generalizability of the findings to other healthcare settings with varying resources and treatment protocols. The focus on 1-year mortality may not capture long-term outcomes, and socioeconomic factors influencing patient outcomes may not have been adequately addressed. Despite these limitations, this study provides valuable insights into the mortality rates among nonagenarians following hip fracture surgery, contributing to the growing body of knowledge in geriatric orthopedics.

### 4.7. Future Research Directions

Future research should focus on validating the GNRI as a dynamic prognostic tool in nonagenarian patients with PFFs. While our study demonstrated its utility upon admission, it remains unclear whether the GNRI changes over the course of hospitalization and whether such changes correlate with clinical outcomes. Prospective studies should evaluate the impact of nutritional interventions on GNRI values and investigate whether improvements in GNRI are associated with better survival and functional recovery. Additionally, determining the optimal timing for GNRI assessment—whether at admission, postoperatively, or during rehabilitation—could enhance its clinical applicability. Such investigations could provide a clearer understanding of how to integrate the GNRI into routine clinical practice to improve outcomes in this vulnerable population. These future studies could address some of the limitations discussed in the following section, particularly regarding the retrospective design and limited assessment of comorbidities.

## 5. Conclusions

This study underscores the importance of identifying and managing specific prognostic factors in nonagenarian patients with PFFs. A low BMI, cognitive impairment, low albumin levels, and lengthy hospital stays were significantly associated with increased mortality rates. By addressing these factors through tailored clinical interventions, healthcare providers can improve the overall survival and quality of care for this vulnerable patient population. These findings highlight the need for a comprehensive multidisciplinary approach to treating older patients with hip fractures. Our study results suggest avenues for further research, including large-scale multicenter studies for generalization, long-term follow-up for deeper prognostic insights, and interventional studies, to improve treatment protocols.

## Figures and Tables

**Table 1:** Patient characteristics.

Characteristic	Value
**Age, years**	93.2 ± 2.8
**Sex**	
Female	242
Male	43
**Fracture type**	
Trochanter	183
Neck	102
**Treatment**	
Compression hip screw	94
Nail	89
Hemiarthroplasty	74
Cannulated cancellous hip screws	28
**Days from injury to surgery**	3.0 ± 4.0
**Days from admission to surgery**	1.9 ± 2.6
**Length of hospital stay (days)**	18.9 ± 9.3
**Follow-up period (months)**	18.2 ± 15.4

Data are presented as *n* or mean ± SD.

**Table 2:** Univariate analysis of factors affecting survival.

Variables	Alive (*n* = 203)	Deceased (*n* = 82)	*p*-Value
**Age, years**	92.8 ± 2.6	94.0 ± 3.1	0.015
**Sex**			0.10
Female	177	65	
Male	26	17	
**BMI, kg/m^2^**	20.2 ± 2.8	18.7 ± 3.3	<0.0001
**Cognitive impairment**			0.0004
Yes	141	73	
No	62	9	
**Fracture type**			0.13
Trochanter	136	47	
Neck	67	35	0.48
**Implant type**			
Dynamic Hip System	69	25	
Intertrochanteric nail	67	22	
Cannulated cancellous hip screws	18	10	
Hemiarthroplasty	49	25	
**Days from injury to surgery**	3.1 ± 4.2	2.7 ± 3.5	0.50
**Days from admission to surgery**	2.0 ± 2.7	1.9 ± 2.4	0.56
**Length of hospital stay (days)**	17 ± 7.8	22 ± 11.7	0.003
**Duration of surgery (minutes)**	82 ± 29.7	80 ± 26.1	0.77
**Hemoglobin**	10.8 ± 1.7	10.7 ± 1.9	0.51
**Albumin**	3.6 ± 0.46	3.3 ± 0.51	<0.0001
**White blood cells**	9.1 ± 3.3	9.0 ± 3.9	0.62
**GNRI**	91.8 ± 9.1	84.9 ± 10.2	<0.0001

Data are presented as *n* or mean ± SD. GNRI: Geriatric Nutritional Risk Index.

**Table 3:** Multivariate analysis of factors affecting survival.

Variables	OR	Interval		*p*-Value
		Lower	Upper	
**Length of hospital stay**	1.05	1.02	1.08	0.001
**Cognitive impairment**	3.08	1.37	6.95	0.007
**GNRI**	0.93	0.90	0.96	<0.001

GNRI: Geriatric Nutritional Risk Index.

## Data Availability

Due to ethical considerations, we are unable to make the full dataset publicly available. However, we are open to discussing requests for anonymized data from qualified researchers under appropriate agreements.
